# Distribution and Functions of TonB-Dependent Transporters in Marine Bacteria and Environments: Implications for Dissolved Organic Matter Utilization

**DOI:** 10.1371/journal.pone.0041204

**Published:** 2012-07-19

**Authors:** Kai Tang, Nianzhi Jiao, Keshao Liu, Yao Zhang, Shuhui Li

**Affiliations:** State Key Laboratory of Marine Environmental Sciences, Xiamen University, Xiamen, China; University of Delaware, United States of America

## Abstract

**Background:**

Bacteria play critical roles in marine nutrient cycles by incorporating and redistributing dissolved organic matter (DOM) and inorganic nutrients in the ocean. TonB-dependent transporter (TBDT) proteins allow Gram-negative bacteria to take up scarce resources from nutrient-limiting environments as well as siderophores, heme, vitamin B12, and recently identified carbohydrates. Thus, the characterization of TBDT distribution and functions is essential to better understand the contribution TBDT to DOM assimilation and its consequences on nutrient cycling in the environment.

**Methodology/Principal Findings:**

This study presents the distribution of encoded known and putative TBDT proteins in the genomes of microorganisms and from the Global Ocean Survey data. Using a Lek clustering algorithm and substrate specificities, the TBDT sequences were mainly classified into the following three groups: (1) DOM transporters; (2) Siderophores/Vitamins transporters; and (3) Heme/Hemophores/Iron(heme)-binding protein transporters. Diverse TBDTs were found in the genomes of oligotroph *Citromicrobium bathyomarinum* JL354 and *Citromicrobium* sp JLT1363 and were highly expressed in the stationary phase of bacterial growth. The results show that the *Gammaproteobacteria* and the *Cytophaga*-*Flavobacterium*-*Bacteroides* (CFB) group bacteria accounted for the majority of the TBDT gene pool in marine surface waters.

**Conclusions/Significance:**

The results of this study confirm the ecological importance of TBDTs in DOM assimilation for bacteria in marine environments owing to a wide range of substrate utilization potential in the ubiquitous *Gammaproteobacteria* and CFB group bacteria.

## Introduction

Bacteria play important roles in ocean carbon and nutrient cycling by incorporating and redistributing dissolved organic matter (DOM) and inorganic nutrients [1], [2]. Transport proteins are a primary mechanism in inorganic nutrient uptake and DOM assimilation in microbial cells. Therefore, the identification and characterization of transport proteins may be important towards understanding a broad range of DOM molecules available for assimilation and utilization by microbes.

Marine bacteria require various efficient transport systems to successfully sequester essential nutrients from ocean environments with limited resources. Bacterial transporters are frequently observed in microbial metatranscriptome and metaproteome data [3]–[8]. Majority of the transporters detected in marine environments include ATP-binding cassette (ABC) transporters [3]–[5], tripartite ATP-independent periplasmic (TRAP) transporters [4], phosphotransferase system (PTS) transporters [4], and TonB-dependent transporters (TBDTs) [6]–[8]. ABC transporters are ubiquitous in bacteria and function in the import of growth substrates or factors, including carbohydrates, amino acids, polypeptides, vitamins, and metal-chelate complexes [9]. ABC transporters are considered as good indicators for bacterial DOM utilization patterns [10]. PTS is another important mechanism used by bacteria for carbohydrate uptake [11], and TRAP transporters transport C4-dicarboxylates malate, succinate, and fumarate [12]. Moreover, TBDT in the bacterial outer membrane often promotes the transport of rare nutrients and is known for its high-affinity uptake of iron complexes (siderophores, citrate, heme, hemoglobin, transferrin, and lactoferrin) [13], [14] as well as of vitamin B12 [15], vitamin B1 [16], and metals (nickel) [17]. In addition, a TBDT can combine with a TonB-ExbB-ExbD system and a specific ABC transporter system to complete the transfer of siderophores or vitamin B12 across the cytoplasmic membrane [18], [19]. Interestingly, several TBDTs are well characterized and are able to access a wide variety of substrates [19]–[24]. Experimental data reveal that carbohydrates, amino acid, and organic acid are TonB-dependent substrates [20]–[24]. For example, TBDTs were proposed to participate in the transport of macromolecules, such as alginate and N-acetyl-chitinoligosaccharides, as observed for *Xanthomonas* bacteria [20], [21], *Sphingomonas* sp. strain A1 [22], and *Caulobacter crescentus* [23]. *Sphingomonadales* organisms are well known for their growth in oligotrophic marine waters and for their ability to degrade aromatic compounds [25]. *Sphingomonas wittichii* RW1 affiliated with *Sphingomonadales* currently contains the most TBDTs among all microorganisms [25]. These TBDTs are used by bacteria for uptake of diverse substrates, including aromatic compounds [25]. The statements above clearly indicate that TBDTs are involved in DOM uptake.

TBDT sequences exhibited low sequence similarity with one another, indicating an unexpectedly high diversity within TBDT sequences. The accounts of the TBDT system in different bacteria were summarized based on bioinformatic analyses [14], [26], [27], and previous research identified the functions of few TBDTs in cyanobacteria and nitrogen-fixing nodulating bacteria by using CLANS [28]. The siderophores-related TBDTs in marine bacteria and metagenomics have recently been analyzed using OrthoMCL [29]. However, previous attempts on the genomic analysis of TBDT were mainly focused on a rather narrow group of species [26], [27] or on a particular subfamily of TBDTs, such as siderophore transporters [14]. A complete functional characterization of TBDT has not been performed to date.

This study performs a comprehensive survey of TBDT in all available bacterial genomes and in the Global Ocean Survey (GOS) metagenome [30]. The predicted TBDTs were classified into groups according to their pair-wise sequence similarities and substrate type. The prediction of substrates for the selected TBDTs was conducted using gene context analysis. Majority of the TBDT sequences in the metagenomic data sets originated from the *Gammaproteobacteria* and *Cytophaga*-*Flavobacterium*-*Bacteroides* (CFB) group bacteria. TBDTs for siderophores and DOM transportation were present in the *Sphingomonadales* organisms as well. The genomes of two marine bacteria, namely, the oligotroph *Citromicrobium bathyomarinum* JL354 [31] and *Citromicrobium* sp. JLT1363 [32] from the order *Sphingomonadales*, were sequenced and annotated, and TBDTs were found to be abundant in their genomes. Moreover, the protein expression profiles of cells show that TBDTs were involved in coping with the low levels of nutrients during the stationary phase. The overall findings have advanced the understanding of the role of TBDTs in oceanic organic matter and nutrient cycles.

## Results

### Distribution of Transporters in Bacterial Genomes

TBDT genes are distributed among a variety of divergent bacterial taxa, including *Proteobacteria*, Cyanobacteria, *Verrucomicrobia*, and *Spirochetes*. Overall, approximately 36% of the draft and completed genomes from NCBI (3,274 genomes) contain TBDTs. The number of TBDT genes in various bacteria is presented in Table S1. This study primarily focuses on TBDTs in marine bacteria, and thus, species were included such that all the major groups of bacteria are covered. As described in Table S2, 68% of the 174 analyzed genomes contained 1–15 TBDTs and 14% contained more than 30 TBDTs. The other transporters of the representatives of the major bacterial groups, including members of the *Sphingomonadales*, SAR11, the *Roseobacter* clade, *Gammaproteobacteria*, CFB and cyanobacteria, which are distributed in the surface marine waters [33], were investigated further (Table 1).

**Table 1:** Comparison of substrate dependent transporters among selected marine bacteria.

Organism	Taxonomic affiliation	Genome Size (Mb)	Total number of ORFs	TBDT*	ABC transporter†	PTS	TRAP	Trophic strategy‡
*Citromicrobium bathyomarinum* JL354	*Alphaproteobacteria-Sphingomonadales*	3.27	3235	27 (8)	22(7)	1	0	N/A
*Citromicrobium* sp. JLT1363	*Alphaproteobacteria-Sphingomonadales*	3.12	3003	31(10)	25 (8)	1	1	N/A
*Erythrobacter litoralis* HTCC2594	*Alphaproteobacteria-Sphingomonadales*	3.05	3011	18 (6)	23 (8)	3	0	extreme oligotrophs
*Sphingomonas wittichii* RW1	*Alphaproteobacteria-Sphingomonadales*	5.92	4850	134 (23)	34 (6)	4	0	extreme oligotrophs
*Roseobacter denitrificans* OCh 114	*Alphaproteobacteria-Roseobacter* clade	4.32	4129	1 (0)	110 (25)	2	22	extreme oligotrophs
*Dinoroseobacter shibae* DFL 12	*Alphaproteobacteria-Roseobacter* clade	4.42	4192	3 (1)	86 (19)	4	28	extreme oligotrophs
*Ruegeria pomeroyi* DSS-3	*Alphaproteobacteria-Roseobacter* clade	4.6	4252	0 (0)	102 (22)	4	26	moderate copiotrophs
*Candidatus* Pelagibacter ubique HTCC1062	*Alphaproteobacteria-*SAR11 clade	1.31	1354	0 (0)	24 (18)	0	3	extreme oligotrophs
*Candidatus* Pelagibacter ubique HTCC1002	*Alphaproteobacteria-*SAR11 clade	1.33	1393	0 (0)	23 (17)	0	2	extreme oligotrophs
*Rhodopseudomonas palustris* CGA009	*Alphaproteobacteria*	5.46	4820	17 (3)	117 (21)	5	8	moderate oligotrophs
*Prochlorococcus marinus* str. MIT 9202	Cyanobacteria	1.69	1890	1 (1)	40 (24)	0	0	extreme oligotrophs
*Prochlorococcus marinus* str. MIT 9301	Cyanobacteria	1.64	1906	0 (0)	33 (20)	0	0	extreme oligotrophs
*Synechococcus* sp. WH 7803	Cyanobacteria	2.37	2533	0 (0)	47 (20)	0	0	extreme oligotrophs
*Synechococcus sp.* WH 8102	Cyanobacteria	2.43	2519	0 (0)	50 (21)	0	0	extreme oligotrophs
*Synechococcus* sp. PCC 7002	Cyanobacteria	3.41	3187	6 (2)	78 (23)	0	0	extreme oligotrophs
*Cytophaga hutchinsonii* ATCC 33406	CFB group bacteria	4.43	3785	5 (1)	42 (9)	0	0	moderate copiotrophs
*Flavobacteria bacterium* BBFL7	CFB group bacteria	3.08	2592	10 (3)	32 (10)	0	1	moderate copiotrophs
*Flavobacterium johnsoniae* UW101	CFB group bacteria	6.1	5017	47 (8)	41 (7)	0	0	moderate copiotrophs
*Flavobacteria bacterium* MS024-3C	CFB group bacteria	1.52	1384	7(5)	16(11)	0	0	N/A
*Polaribacter* sp. MED152	CFB group bacteria	2.97	2611	10(3)	22(7)	0	0	N/A
*Salinibacter ruber* DSM 13855	CFB group bacteria	3.59	2801	16 (4)	59 (16)	0	3	moderate copiotrophs
*gamma proteobacterium* HTCC2207	*Gammaproteobacteria-*SAR92 clade	2.62	2388	17 (6)	21 (8)	2	1	extreme oligotrophs
*gamma proteobacterium* NOR5-3	*Gammaproteobacteria-*NOR5/OM60 clade	4.20	3671	27 (6)	37 (9)	2	2	extreme oligotrophs
*gamma proteobacterium* HTCC2143	*Gammaproteobacteria-*BD1-7 clade	3.93	3662	22 (6)	39 (10)	2	2	extreme oligotrophs
*gamma proteobacterium* HTCC2148	*Gammaproteobacteria-*NOR5/OM60 clade	4.31	3827	37 (9)	49 (11)	2	2	extreme oligotrophs
*gamma proteobacterium* HTCC2080	*Gammaproteobacteria-*NOR5/OM60 clade	3.58	3185	28 (8)	17 (5)	2	1	extreme oligotrophs
*Congregibacter litoralis* KT71	*Gammaproteobacteria-*NOR5/OM60 clade	4.33	3941	26 (6)	27 (6)	2	2	extreme oligotrophs
*Pseudoalteromonas haloplanktis* TAC125	*Gammaproteobacteria-Alteromonadales*	3.85	3485	34 (9)	29 (8)	4	0	moderate copiotrophs
*Idiomarina loihiensis* L2TR	*Gammaproteobacteria-Alteromonadales*	2.84	2628	28 (10)	29 (10)	4	0	moderate copiotrophs
*Shewanella baltica* OS185	*Gammaproteobacteria-Alteromonadales*	5.31	4394	33 (6)	53 (10)	7	3	extreme copiotrophs
*Alteromonadales bacterium* TW-7	*Gammaproteobacteria-Alteromonadales*	4.10	3783	32 (8)	42 (10)	4	0	N/A
*gamma proteobacterium NOR51-B*	*Gammaproteobacteria*	3.26	2930	32(10)	44(13)	4	3	N/A
*Nitrosococcus oceani* ATCC19707	*Gammaproteobacteria*	3.52	3017	7 (2)	34 (10)	5	1	moderate oligotrophs
*Vibrio vulnificus* YJ016	*Gammaproteobacteria*	5.21	5098	7 (1)	95 (18)	24	9	extreme copiotrophs
*Photobacterium profundum* SS9	*Gammaproteobacteria*	6.40	5489	7 (1)	84 (13)	5	10	extreme copiotrophs
*Acinetobacter baumannii* ATCC17978	*Gammaproteobacteria-Acinetobacter*	4.00	3367	22(6)	34(9)	2	0	moderate oligotrophs
*Acinetobacter sp.* ADP1	*Gammaproteobacteria-Acinetobacter*	3.60	3307	22(6)	38(11)	3	0	moderate oligotrophs
*Nitrosomonas europaea* ATCC 19718	*Betaproteobacteria*	2.81	2461	30 (11)	43 (15)	4	0	moderate oligotrophs
*Ralstonia eutropha* H16	*Betaproteobacteria*	4.05	3651	17 (4)	65 (16)	6	5	moderate copiotrophs

Abbreviations of transporters are: TBDT, TonB-dependent transporter; PTS, phosphotransferase system; TRAP, tripartite ATP-independent periplasmic protein.

* The number of transporters per Mbp of each genome is shown in bracket(s).

† The ABC transporters related multiple domains encode as one polypeptide.

‡ Information from reference [34].

N/A means no data.

TBDT sequences appeared to be highly prevalent in the orders *Sphingomonadales*, *Alteromonadales*, in the CFB group bacteria, and in the oligotrophic marine *Gammaproteobacteria* (OMG) group, which includes members of the BD1-7, SAR92, and OM60/NOR5 clades (Table 1). *Citromicrobium bathyomarinum* JL354 and *Citromicrobium* sp. JLT1363 contain 27 and 31 TBDT sequences in their genomes. Except for strain *Ruegeria pomeroyi* DSS-3, which lacked TBDT sequences in the genome, all the other analyzed *Roseobacter* strains carried one to five TBDT sequences (Table S1). The genes for TBDT were also present in several cyanobacterial strains, and not all marine bacteria contained TBDT. For example, *Candidatus* Pelagibacter ubique, which is the most abundant prokaryote in the SAR11 clade, did not contain TBDT.

However, ABC transporters were recognized in all the analyzed genomes, indicating that they are absolutely essential for substrate transport in bacteria. The number of ABC transporter genes ranged from 22 to 117 genes per genome (Table 1), and the number of ABC transporters was higher than that of TBDTs in most bacteria. No clear correlation between the number of transporters and bacterial trophic strategy [34] or genome size was found because many copiotrophic or oligotrophic bacteria had an unusually high number of genes that encode TBDTs or ABC transporters in their genomes. However, the number of ABC transporters per Mbp of each genome was distinctly higher in some bacteria with few TBDTs. For example, a specific enrichment in the number of ABC transporters was present in the *Roseobacter* clade, and SAR11 and cyanobacteria had abundant ABC transporters (Table 1). A significant negative correlation between the number of ABC transporters and TBDTs per Mbp of each genome was found (Spearman rank correlation: *r*_s_ = −0.677; N = 39; *P*<0.0001). The *Roseobacter* clade and *Vibrio* had an unusually high number of genes that encode TRAP transporters and PTS systems in their genomes, respectively. The distribution of transporter genes in bacterial genomes suggests that the use of the transporter system among bacteria had considerable differences. However, the presence of a reasonable number of transporters enabled the bacteria to use a broad range of substrates for growth.

### Distribution of Transporters in Aquatic Systems

The GOS dataset used in this study contained genomic sequences derived from 57 samples from the open ocean to the coast across temperate and tropical regions as well as few non-marine aquatic samples [30], [35]. A total of 44073 putative TBDT homologs that varied in number from 65 to 3,946 were found in the samples (Table S3). The frequency of the TBDTs in the total open reading frames (ORFs) provided a rough estimate of the prevalence of TBDT in ocean waters, ranging from a low of approximately 0.1% (GS4) in the North American East Coast to a high of approximately 2% (GS18) in the Caribbean Sea Coastal (Table S3). To compare the distribution of TBDTs in diverse environmental samples, the frequency of TBDT was determined by comparing the average number of control single-copy gene hits for each site as an indicator to be used for the estimation of the total genome equivalents (see *Materials and Methods*). The Caribbean Sea Coastal GS18, which has extremely high frequency, was the most abundant station. The Sargasso Sea data sets (GSb, GSc, and GS1a) and the data sets from the Galapagos Islands (warm seep GS30 and GS31) also exhibited high hits, but with moderate frequency. Moreover, the estimations of frequency and number of genes suggested that TBDTs were less common in the Indian Ocean, with the exception of GS117, GS110b, and GS122b, as well as in the coastal and estuarine stations of the North American East Coast (GS4, GS11 and GS12) and reef stations (GS49 and GS51) (Table S3). No significant relationships between the environmental factors (chlorophyll *a*, temperature, and salinity) and TBDT distributions were found. Moreover, a greater number of ABC transporters with extremely high frequency were found at hypersaline site GS33 than at the other sites (Table S3). The relatively high number and frequency of ABC transporter gene hits were identified in the warm seeps GS30 and GS31. The open ocean sites (Sargasso Sea GS1a, Indian Ocean GS110b, and GS112b) as well as reef station GS108b appeared to show relatively low frequency. No clear trend was observed in the type of environment in which the TBDT genes or ABC transporter genes were observed frequently. A significant positive correlation between the amounts of TBDT and ABC transporter homologs (Spearman rank correlation: *r*_s_ = 0.791; N = 57; *P*<0.0001) seemed to exist. Most of the observed frequencies of the ABC transporter homologs in the total ORFs along the GOS transect were approximately 1% (Table S3).

### Functional Characterization of TBDTs in Marine Bacterial Genomes

In this study, the Lek clustering algorithm was used to cluster the GOS sequences and NCBI sequences that contain TBDT sequences of known functions and investigate any sequence homology between TBDTs with known functions and the various putative ones (Table 2). Notably, the clustering results were not influenced by the removal of the GOS sequences.

**Table 2:** Functional classification of the TBDT sequences extracted from NR and the GOS datasets.

Function categories	Lek Cluster number	TBDT*	Substrates†	NCBI sequences	GOS peptides
**Group I: DOM transporters**	**Cluster 3090**	Sden_2708(91794059), CPS_1021(71281574), PSPTO_3242(28870407), XCC2385(21231822), MalA(220964479), XCC2469(21231904), MalA(16126526), NagA(13421615), XCC2828(21232259), SO_3514(24375018), CC_0446(16124701), XCC0120(21229598), XCC2944(21232375), XCC4120(21233542) XCC2887(21232318)	Chito-oligosaccharides, phytate, maltodextrin, maltose, chitin, xylan, xylose, pectin	816	3107
	**Cluster 720**	RagA(110636966, 110636973),SusC(29349110), CsuF(29348741)	Digested proteins, starch/malto-oligo-saccharides, chondroitin sulfate/hyaluronic acid	3565	2060
	**Cluster 427**	XCC4222(21233639)	Arabinose	945	5748
	**Cluster 952**	SuxA(21232787), Sfri_3988(114565138)	Sucrose	53	4
**Group II: Siderophores/Vitamins transporters**	**Cluster 3303**	FecA(729471, 16132112)	Ferric-citrate	288	946
	**Cluster 410**	FmtA(53719389), PupA(45723), FatA(132510), FcuA(1169655), ViuA(267356), RhtA(6685883), BfrI(33592999), Bcep18194_b2436(78063283), PupB(585759), IutA(84060860), SftP(6019468), XCC0674(21230149), CPS_0067(71281279), FhuE(16129065), PbuA(1172035), FpvA(12230910), FauA(4589285), FhuE(2507465), FptA(1169730), SO_2715(24374256), PhuR(3044098), PrhA(17549099), IutA(1170593),FctA(871032), BfrZ(6850914), FyuA(517234),BauA(49175779), Fiu(170080464), FhuA(16128143), VciA(147673813), PiuA_Fiu(115587765), FoxA(1169726), FegA(1518696),FhuA(2507464), OptS(116050410), IrpC(17380443), OrbA(76810798)	Aerobactin, alcaligin, anguibactin, catecholates, chrysobactin, coprogen, ferrioxamine B, rhodoturolic acid, desferrioxamine, ferric malleobactin, ferric ornibactin, ferrichrome, hexylsulfate, pseudobactin A, pseudobactin M114, pyochelin, pyoverdine, rhizobactin 1021, thiamin, vibriobactin, yersiniabactin	4576	9462
	**Cluster 973**	XCC3067(21232497), PA1271(15596468), RS02718(17547119), VC0156(15640186), BtuB(416728), CC1750(109897435), BPSL0976(53718618), Saro0693(23107401), CirA(2507462,16130093), FepA(16128567), PirA(2981053), PfeA(548479), FepA(2507463), IroN(2738252), CfrA(112360090),IrgA(12644182), BfrA(1314835), BfeA(538279), RSP_2402(77462960)	Vitamin B12, catecholates,enterobactin, 2,3-dihydroxybenzoylserine(DHBS),	1677	1082
	**Cluster 325**	SO_0815(24372404)	Vitamin B12	168	91
	**Cluster 180**	BF1991(53713281), BF0615(53711906), PG1899(34541505)	Fibronectin, thiamin	113	1690
	**Cluster 2835**	GOX1347(58039791)	Thiamin	100	6
**Group III: Heme/Hemophores/Iron(heme)-binding transporters**	**Cluster 1609**	HumA(53829495)	Heme	413	1432
	**Cluster 1856**	HxuC(1170441), PfhR(4838477), HgbA(28194090), HemR(3646475,6016198), HgbA(33152990), HpuB(11386826), HasR(34787214), HmbR(687640), HmuR(2501236), ChuA(1763009), ShuA(1655877), VctA(18476494), ShuA(82778670), TbpA(150361), FrpB4(15646121), HuvA(12697532), TdhA(33151615), BLL7076(27382187), LbpA(915278), HutA(529727, 148292078), HutR(147671724)	Heme	1244	515
**Group IV: Metal transporters**	**Cluster 767**	NosA(151399), OprC(1498191,15598985),	Copper, Copper chelate	184	110
	**Cluster 987**	Bll6948(27382059), Daro_3944(71909555), RPA_4757(39937815),pHCG3_081(47177035), Daro_1684(71907314)	Nickel, Cobalt	64	0
			Total number of sequences clustered together with the identified substrate dependent TBDTs	14206	26253
			Total number of TBDTs	15905	44073

* Standard gene or protein identificators of experimentally characterized TBDT and of TBDT with predicted substrate retrieved from GenBank based on References [26] and [27]. Designated numbers are given in brackets.

† A list of the corresponding substrates for TBDT.

The results found 3,343 clusters of TBDTs in the bacterial and environmental sequences, in which 17 Lek clusters that contain the experimentally determined TBDT sequences were found (Table 2). The Lek clusters were subsequently classified into several groups (I to IV) according to the known substrate types (“DOM,” “siderophores/vitamins,” “heme/hemophores/iron(heme)-binding proteins,” and “metals”). Each group contained sequences from two or more Lek clusters (Table 2). Group I, which was recognized in the current analysis, consisted of novel transporters for various types of DOM, including carbohydrates, amino acids, lipids, organic acid, and protein degradation products. Members of Group II include siderophores, vitamin B1, and vitamin B12. Group III consisted of TBDTs that transport iron from heme or iron proteins with high affinity. Nickel and cobalt were the predicted substrates in Group V. The amino acid sequences of clustered siderophores-related transporters were not significantly similar to the TBDTs involved in DOM uptake. However, some of siderophores-related transporters shared some sequence homologies with the vitamin B12/vitamin B1 transporter proteins and were thus clustered together (Table 2).

TBDTs in selected marine bacterial genomes mainly functioned as transporters with diverse substrates, as summarized in Table S2. Members of clusters 427 and 3090 (DOM transporters) were mainly found in *Gammaproteobacteria*, including *Alteromonadales* (such as *Alteromonadales bacterium* TW-7) and the OMG group of bacteria (such as *gamma proteobacterium* HTCC2207), as well as in *Sphingomonadales* (such as *Citromicrobium bathyomarinum* JL354 and *Citromicrobium* sp. JLT1363). Cluster 720 (DOM transporters) was specific in its distribution to species of the CFB group of bacteria. One or two corresponding genes were identified in most of the analyzed CFB species. As many as 46 and 49 genes were found in *Pedobacter heparinus* DSM 2366 and *Pedobacter* sp. BAL39, respectively (Table S2).

Members of cluster 410 (siderophore transporters) were found in the genomes of most of the species analyzed using TBDT. Majority of the siderophore transporters came from the gamma- and alpha-proteobacteria (such as the *Alteromonadales*, the OMG group of bacteria, and the *Sphingomonadales*). As many as 117 genes that encode siderophore transporters were found in *Sphingomonas wittichii* RW1 (Table S2).

Clusters 1609 and 1856 contained TBDT that transports iron from heme or iron-binding protein as an alternative iron source for the bacteria. The corresponding genes were identified in most of the analyzed species, and their number ranged from one to four genes per genome (Table S2). TBDTs in the *Roseobacter* clade and cyanobacteria were mainly distributed in clusters 410 and 1856, suggesting that they were responsible for iron acquisition in the bacteria. The members of the last group (clusters 767 and 987) remained as few sequences, indicating that nickel- or cobalt-specific TBDTs were not common among the bacteria (Table S2).

### Functional Characterization of TBDTs in Metagenomes

**Figure 1:**
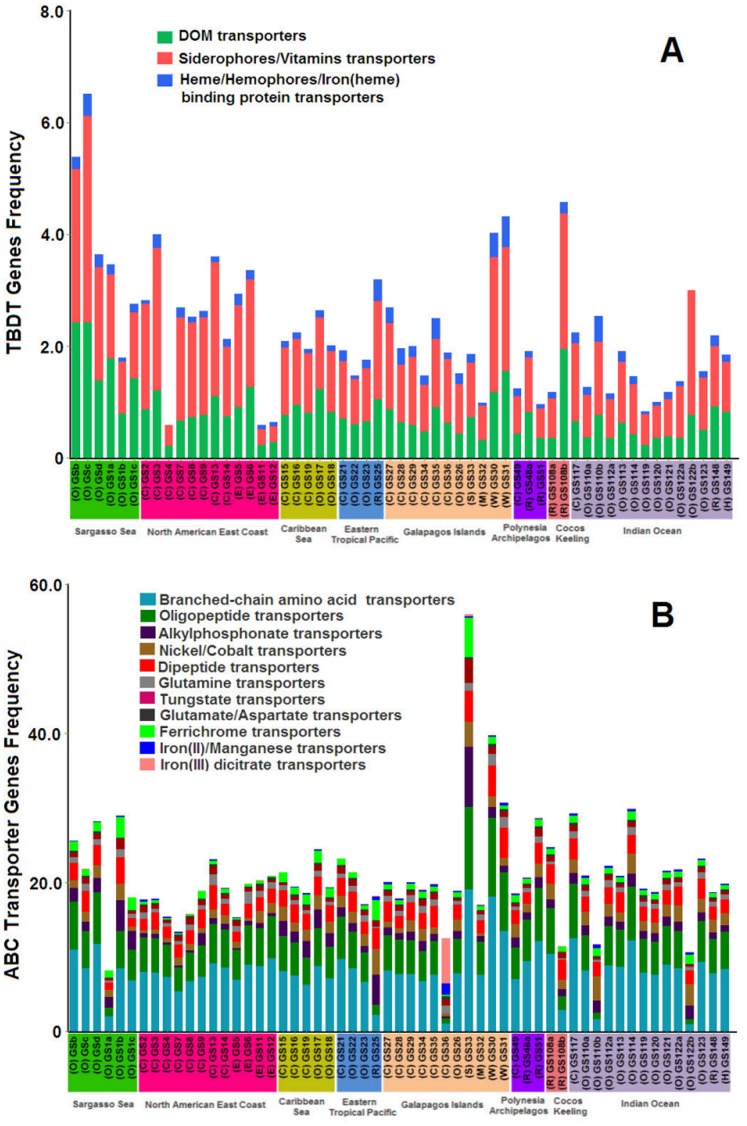
Frequencies of TBDT (A) and ABC transporter (B) genes in GOS samples at different locations. The number of transporters from GOS sites was normalized as described in the Methods section. The frequency is relative to the average number of six single-copy gene hits for each site. A color scheme is used to show the functional groups. Color (*bottom axis*) represents different sampling areas and each habitat type (n) is indicated (O: open sea, C: coastal, E: estuary, R: reef, W: warm seep, S: hypersaline, H: harbor, M: mangrove). Samples 01a, 01b and 01c were obtained using different size fractions from the same station: sample 01a, 20-3 µm; sample 01b, 3-0.8 µm; sample 01c, 0.8-0.1 µm.

**Figure 2:**
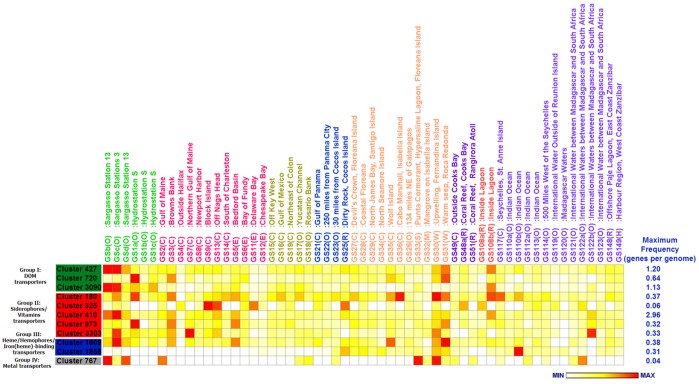
Heat map comparison of frequencies each cluster in different GOS samples. The frequency value each GOS site was assigned a color relative to the maximum value seen in each cluster. The maximum value of frequency is shown at the end of its corresponding row. The colors represent 0 (white) to MAX (red). The different GOS sampling areas are highlighted using colored text.

Similarly, the TBDT genes with varying frequencies in all metagenomic datasets were mainly for siderophores/vitamin transporters and DOM-related transporters, followed by heme/hemophores/iron(heme)-binding protein transporters (Figure 1A). Nickel and cobalt TBDTs appeared to be uncommon in the surface ocean. Overall, DOM and siderophore transporters were particularly prevalent across 57 GOS sampling sites. High-frequency DOM-related TBDTs from *Gammaproteobacteria* (clusters 427 and 3090) and siderophores-related TBDTs (cluster 410) can be found in the Sargasso Sea (GSb and GSc), as shown in Figure 2. The relatively high-frequency DOM-related TBDTs from the CFB group (cluster 720) were distributed in GS1a, GS3, GS31, and GS35 (Figure 2). In addition, GS31 and GS35 contained heme transporters (cluster 1609) at relatively high frequency and siderophore transporters at relatively moderate frequency, suggesting complementary pathways for acquiring iron (Figure 2).

Predicted substrates for the frequently abundant ABC transporters in marine environments branched into chain amino acids, oligopeptides, alkylphosphonates, dipeptides, tungstates, glutamates, and aspartates (Figure 1B). An overlap between the TonB-dependent substrates and the ABC transporter substrates, such as nickel, cobalt, and iron complex, was observed. The ABC transporter specificity for various amino acids and peptides was notably prevalent across marine environments, suggesting that the ABC transporters performed essential roles as nitrogen resources in the bacterial uptake of amino acids or peptides. The Fe^3+^ ABC transporter genes for ferrichrome and iron dicitrate were more abundant than the Fe^2+^ transporter genes in the metagenomes (Figure 1B), as also indicated in previous studies [14]. However, the overall frequency of the identified siderophores-related TBDT genes (estimated average frequency of approximately 0.8) was close to that of the Fe^3+^ ABC transporter genes (estimated average frequency of approximately 1.1). Thus, the Fe^3+^ TonB-dependent transportation strategy may be more prevalent than originally believed [14].

**Figure 3:**
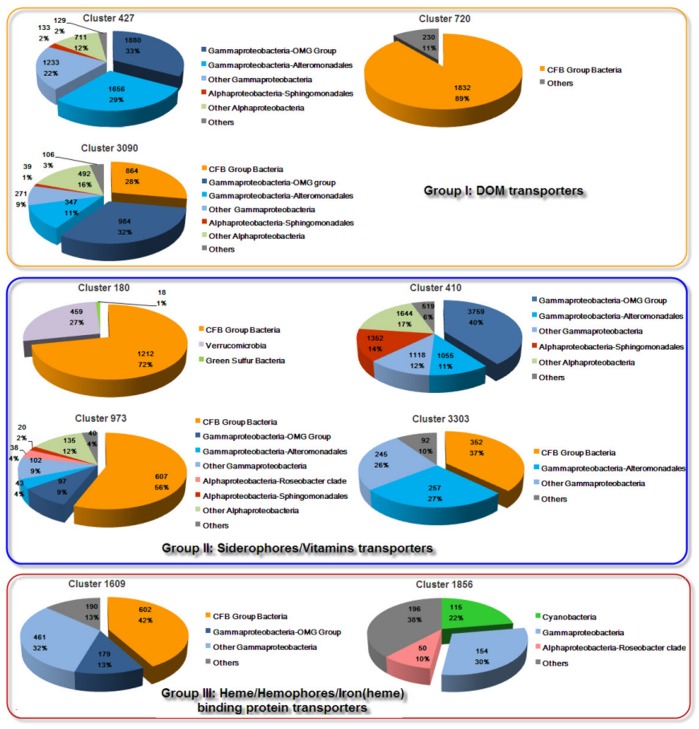
Best BLAST matches in GenBank of TBDT sequences along the GOS transect and their taxonomic affiliation. Colors are used to represent the major phylogroups.

The retrieved GOS sequences were dominated by TBDT genes that were closely related to *Gammaproteobacteria* (primary members of the *Alteromonadales* and the OMG group of bacteria) with moderate contributions from species in the CFB phyla (Figure 3). Sequences related to the order *Sphingomonadales* were generally rare, but were also contributors to siderophores- and DOM-related transporters in the metagenomic data (Figure 3). Taxonomic distribution of the retrieved GOS sequences in the major protein families was similar to the TBDT distribution pattern in the bacterial genome.

The stringent E-value cutoff values for the BLASTP search [36] and the Lek clustering [37], [38] were chosen to ensure that the functionally relevant TBDTs are present in the same cluster when the predicted clusters were too high. Approximately 11% of the TBDTs from the non-redundant (NR) database and 40% of the TBDTs from the GOS were categorized in the clusters without function-known genes (Table 2). For example, many sequences from GS18 were in the clusters that only contained GOS sequences, although the highest number of TBDTs was observed in GS18 (Table S3). The GOS sequences possibly came from uncultured marine bacteria, which showed relatively low similarity to TBDT sequences from the bacterial genome. On the other hand, TBDT subfamilies that only included GOS sequences can be considered likely to be as-yet undiscovered TBDT subfamilies. The current data supported the notion that the number and variety of TonB-dependent substrates were underestimated [19] and especially that the experimental studies for identifying TBDT substrates in marine samples were extremely limited. The large diversity and structural complexity of DOM in the marine environment make it a potential substrate for TBDTs.

### Genomics-based Prediction of Substrates for TBDTs in the Genomes of Two *Citromicrobium* Strains

The genomic localizations of the genes of the identified TBDTs were analyzed to predict their substrate preferences. *Citromicrobium bathyomarinum* JL354 and *Citromicrobium* sp. JLT1363 are closely related with each other phylogenetically, and both strains have similar functional distribution of TBDT. However, the genomic organization and gene context surrounding the TBDT gene in the bacteria were often not conserved, suggesting the diversity of the substrates for TBDT (Figures S1 and S2). Most of the coding genes for TBDT were not randomly distributed across the genome, but were located in a physiologically meaningful genomic context.

**Figure 4:**
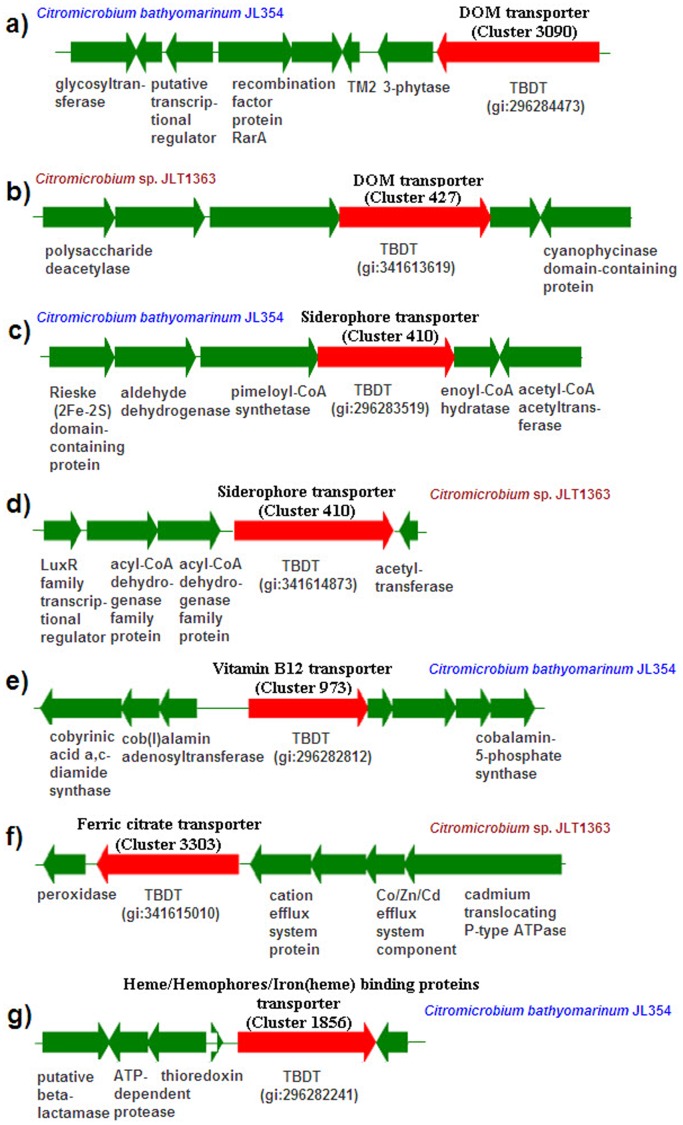
Open reading frames identified in the genome of *Citromicrobium bathyomarinum* JL354 and *Citromicrobium* sp. JLT1363 adjacent to the TBDT gene (marked in red) and predicted to be related to substrate utilization. The arrows indicate the direction of transcription.

Based on the clustering analysis (Figures S1 and S2), majority of TBDTs were found to transport siderophores and DOM. Interestingly, some TBDT genes resided within operons predicted to code for enzymes that transfer fatty acyl substitution; two examples are shown in Figures 4a and 4b. These genes may have essential functions in the production and attachment of the siderophore fatty acyl chain [39]. The siderophore contains a fatty acyl chain or an α-hydroxy carboxylic acid moiety that dominates the marine siderophores [40]. The present findings suggest that *Citromicrobium* bacteria with a fatty acyl side chain are capable of transporting siderophores. Figure 4c and 4d show the other iron transporters. Aside from putative TBDT for heme and siderophores uptake, both strains carried TBDT sequences that may be used for DOM and vitamin B12 import (Table S2). For example, a TBDT gene in *Citromicrobium bathyomarinum* JL354 was adjacent to a phytase gene, suggesting that phytic acid is a substrate for TBDT (Figure 4e). A TBDT in *Citromicrobium* sp. JLT1363 was located in close proximity to a gene-encoding cyanophycinase that degrades the amino acid polymer cyanophycin, which is an important intracellular nitrogen-storer of cyanobacteria (Figure 4f). *Citromicrobium bathyomarinum* JL354 had a gene encoding TBDT proximity to genes associated with vitamin B12 biosynthesis, allowing it to be annotated as a vitamin B12 transporter (Figure 4g).

### Genomics-based Prediction of Substrates for Selected TBDTs from Metagenomes

Overall, the environmental TBDT sequences in the major clusters that were most abundant were best matches to the *Gammaproteobacteria* or CFB group bacteria. Among the species, the *gamma proteobacterium* HTCC2207, *gamma proteobacterium* HTCC2143, *gamma proteobacterium* NOR5-3, *Pedobacter heparinus* DSM 2366, and *Flavobacteria bacterium* MS024-3C species contained the greatest number and greatest identity of recruited GOS sequences in major clusters.

**Figure 5:**
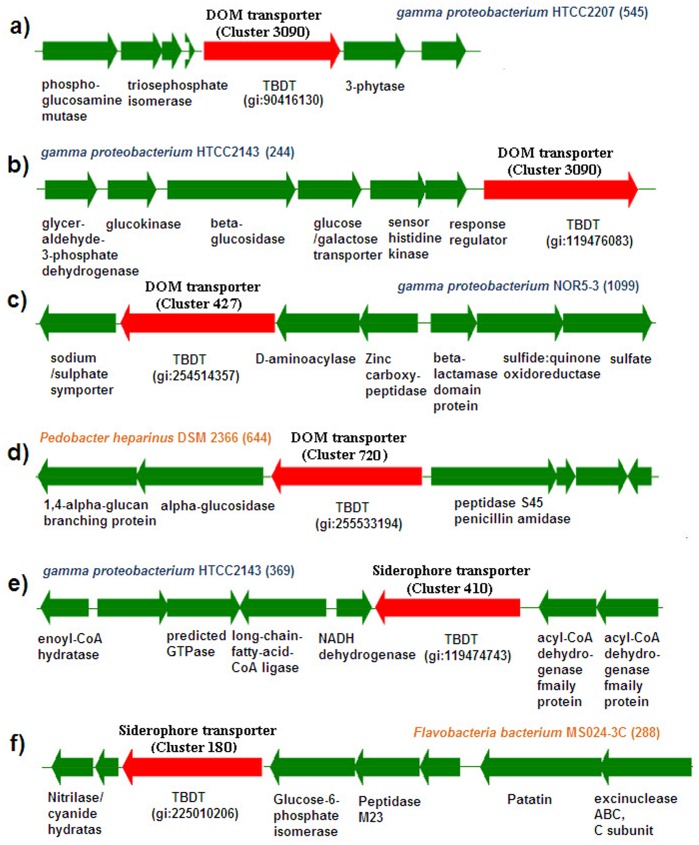
Genomic context of the representative gene clusters containing TBDT (marked in red) from *Gammaproteobacteria* and CFB best matches by environmental sequences in large mounts. The number of the best hits for this gene in GOS is indicated in a bracket.

Figure 5 shows the recruitment sequences observed in large amounts and similar to TBDT genes. Many environmental TBDT sequences in cluster 3090 showed greatest similarity to a phytatic acid transporter from *gamma proteobacterium* HTCC2207 (Figure 5a) or a TBDT gene in *gamma proteobacterium* HTCC2143 located upstream of the galactose utilization genes (Figure 5b). Environmental TBDT sequences in cluster 427 showed the greatest similarity to a TBDT gene in *gamma proteobacterium* NOR5-3 located adjacent to a D-aminoacylase gene that catalyses N-acyl-D-amino acid derivates (Figure 5c). Homologs of a peptidoglycan transporter in *Pedobacter heparinus* DSM 2366 were detected frequently in the environment (Figure 5d). In cluster 180, numerous sequences were homologous to a TBDT in *gamma proteobacterium* NOR5-3 located near a predicted gene encoding cyanide hydratase (Figure 5e). A previous study indicated that a putative siderophore component was involved in cyanide utilization [41]. *Gammaproteobacterium* HTCC2143 had a siderophore transporter gene residing within an operon that was predicted to code for enzymes involved in fatty acid metabolism, similar to the siderophore transporter gene in *Citromicrobium bathyomarinum* JL354 (Figure 5f).

In this study, the results of the genome context analysis suggest that the substrates for TBDTs included siderophores, ferric citrate, vitamin B12, heme, phytic acid, galactose, cyanophycin, N-acyl-D-amino acid derivates, peptidoglycan, and cyanide (Figures 4 and 5). To further detail the DOM utilization patterns, further study must be undertaken to identify more substrates for TBDT, especially from a representative set of bacteria (*Gammaproteobacteria* and the CFB group).

### Proteomic Analysis of *Citromicrobium* Strains

Proteomic views of *Citromicrobium bathyomarinum* JL354 and *Citromicrobium* sp JLT1363 during the stationary phase of culture are presented in Table S4 and Table S5, respectively. Many known abundant bacterial core proteins [42], such as ATP synthase, elongation factors, chaperonin, transporter systems, and ribosomal proteins, contain abundant proteins. The functional categories in the identified proteins are shown in Figures S3 and S4. Transporter activity in terms of numbers was one of the most represented biological process or molecular function gene ontology (GO). Both strains were shown to produce a large number of proteins involved in the TonB-dependent transport system, which comprises a considerable part of the total periplasmic proteome (Table 3). Eight TBDTs and three ABC transporter subunits were detected in a total of 18 periplasmic proteomes from *Citromicrobium bathyomarinum* JL354 (Table 3). Fifteen TBDTs and six ABC transporter subunits were found in a total of 31 periplasmic proteomes from *Citromicrobium* sp JLT1363 (Table 3). These TBDTs might be involved as DOMs, siderophores, or thiamin transporters. Moreover, remarkably high expressions of various TBDTs were observed for *Sphingomonas* [43] and *Pseudoalteromonas haloplanktis* TAC125 [44], which contained a high number of TBDT genes in their genomes. ABC transporters were absolutely required for all bacterial growth. For instance, the ABC transporters were among the most highly detected proteins from the stationary phases of *Ruegeria pomeroyi* DSS-3 [45] and *Candidatus* Pelagibacter ubique [46]. The enrichment of TBDTs in the proteome indicated that TBDT proteins might indeed contribute to bacterial growth. More importantly, TonB-dependent transporter systems may partially compensate for a low number of ABC transporters in marine microorganisms.

**Table 3:** Most abundant transporter proteins detected in in *Citromicrobium bathyomarinum* JL354 and *Citromicrobium* sp. JLT1363, respectively.

Strain	Accession number	Description	Predicted substrate*
***Citromicrobium bathyomarinum*** ** JL354**	**TBDT**		
	gi|296282297	TonB-dependent receptor-like protein	Thiamin
	gi|296282571	putative outer membrane receptor for iron transport	Siderophore
	gi|296282704	TonB-dependent receptor	DOM
	gi|296283519	TonB-dependent receptor	Siderophore
	gi|296284195	TonB-dependent receptor	Siderophore
	gi|296284565	hypothetical protein CbatJ_13101	DOM
	gi|296284567	TonB-dependent receptor	DOM
	gi|296284689	TonB-dependent receptor	Siderophore
	**ABC transporters**		
	gi|296283772	ABC-type transport system	N/A
	gi|296284204	putative ABC transporter ATP-binding protein	N/A
	gi|296284590	ABC-type transport system periplasmic component	N/A
***Citromicrobium*** ** sp. JLT1363**	**TBDT**		
	gi|341613545	TonB-dependent receptor	Hemin/Hemophores/Iron(Heme) binding proteins
	gi|341613619	TonB-dependent receptor	DOM
	gi|341613721	TonB-dependent receptor	Siderophore
	gi|341614223	TonB-dependent receptor, plug	Siderophore
	gi|341614279	TonB-dependent receptor	Siderophore
	gi|341614547	TonB-dependent receptor	Siderophore
	gi|341614682	TonB-dependent receptor	DOM
	gi|341614873	TonB-dependent receptor	Siderophore
	gi|341615010	putative outer membrane receptor protein	Ferric citrate
	gi|341615076	TonB-dependent receptor	DOM
	gi|341615079	TonB-dependent siderophore receptor	Siderophore
	gi|341615951	putative TonB-dependent receptor	Siderophore
	gi|341615953	hemin receptor	Hemin/Hemophores/Iron(Heme) binding proteins
	gi|341616139	TonB-dependent receptor	Siderophore
	gi|341616145	Outer membrane protein	Siderophore
	**ABC transporters**		
	gi|341613400	ABC transporter, ATP-binding protein	N/A
	gi|341614549	iron compound ABC transporter, permease protein	Siderophore
	gi|341614550	ferrichrome ABC transporter ATP-binding protein	Siderophore
	gi|341614655	ABC-type transport system periplasmic component	N/A
	gi|341615504	phosphate transporter ATP-binding protein	phosphate
	gi|341615777	putative ABC transporter ATP-binding protein	N/A

* Predicted substrate based on the functional classification of the TBDT and gene context analysis.

## Discussion

The results of the analysis of the genetic contexts near the TBDT show that some TBDT genes were closely associated with the carbohydrate, amino acid, or other substrate metabolism enzymes in the operons (Figures 4 and 5), suggesting that these genes are functionally linked and may therefore play important roles in diverse DOM uptake for marine bacteria, but are not limited to iron and vitamin B12 uptake only.

Marine organic matter was thought to originate primarily from phytoplankton production [47], [48]. Carbohydrates produced by phytoplankton form an important fraction of the DOM in the ocean [48]. For the *Gammaproteobacteria* and the CFB group of bacteria, TBDTs located in the gene cluster related to carbohydrate metabolism might play a major role in utilizing labile substrates, such as xylose, arabinose, and galactose derived from phytoplankton. In the surface ocean, the ability of monosaccharide incorporation by bacteria depends on ABC transporters because of the abundance and omnipresence of the bacterioplankton population (such as SAR11 and the *Roseobacter* clade) in the marine ecosystem [10]. Carbohydrate utilization of bacterial population is further enhanced in the presence of TBDTs from *Gammaproteobacteria* and CFB bacteria.

Polysaccharides released by phytoplankton were identified as the major constituents of naturally occurring marine high-molecular-weight DOM [49]. Complex molecules that contain N-acetylglucosamine (GlcNA), such as chito-oligosaccharides, are substrates for TBDT [21], [23]. For instance, some CFB can transport and consume chitin and GlcNA within the DOM pool, and thus, it should be a relevant part of chitin-like DOM degradation in marine systems [50]. The N-acyl-D-amino acid derivates may also be used as a putative substrate for TBDT from *Gammaproteobacteria*. Other polysaccharides, such as pectin and alginates, which were the dominant components in diatom and brown algae cell walls, respectively, were also identified as potential substrates for TBDTs [26]. These data suggest that members of the CFB and *Gammaproteobacteria* not only contained polysaccharide hydrolases, but also hydrolysate specific-TBDTs with a selective advantage over other heterotrophic bacteria in a marine environment enriched with polysaccharides, such as in biofilm. The sea surface microlayer has commonly been thought to be a gelatinous biofilm and has been shown to be dominated by *Gammaproteobacteria* and *Bacteroidetes* [51], [52]. A similar result was observed in the biofilm from the Irish Sea [53]. On the other hand, major storage products in phytoplankton, such as cyanophycin in cyanobacteria and starch, were also found to be potential substrates for TBDTs in some bacteria. This observation explains in part the ability of *Gammaproteobacteria* and CFB to consume a wide range of phytoplankton-derived DOM.

DOM also originates in part from the degradation of terrestrial plant materials, such as phytic acid, which is transported to the open sea through rivers [54]. Phytic acid can be an abundant source of phosphorus and carbon for bacteria in coastal waters and is it also used for chelating Fe [54]. Phytic acid-specific TBDT sequences were abundant in the metagenomic data.

Aside from the prevalence of TBDT sequence from (meta-) genomic data, the other ‘omic’ datasets including (meta-) proteomics and (meta-) transcriptomics suggested that TBDTs are physiologically and ecologically significant. The metaproteome sampled from South Atlantic surface waters showed that TBDTs were predominant in membrane proteins, wherein the *Gammaproteobacteria* and CFB group bacteria were the most abundant from the open ocean to the coastal seawater [6]. These TBDT proteins were closely related to *Gammaproteobacteria* and members of the CFB, *Alphaproteobacteria*, and *Deltaproteobacteria*. TBDTs were detected in abundance in the metaproteomic samples from the upwelling in the South China Sea off Vietnam, where the *Alteromonadales* represented the abundant taxa in this research (unpublished data). Transcripts associated with TBDT were significantly overrepresented in the high-molecular weight DOM treatment from the new dominant *Alteromondales* bacteria, whereas *Pelagibacter* and *Prochlorococcus* decreased in relative abundance [7]. TBDTs also comprise the most abundant group of transport-related transcripts within the CFB group bacteria sequences in natural environments [8].

However, ABC transporter transcripts dominated the acquisition of DOM monomers, with minor contributions from the TRAP in the southeastern U.S. coastal seawater wherein the pelagic microorganism *Roseobacter* and SAR11 are dominant [4]. Carbohydrate-related ABC transporter transcripts from *Roseobacter* bacteria accounted for more than 30% of all the DOM-related transporter sequences in the coastal ecosystem [4], suggesting that they outcompeted other marine bacteria for carbohydrates. SAR11 bacteria typically contributed to a significant fraction of amino acid assimilation in surface waters [55]. In the Sargasso Sea, the periplasmic compounds of ABC transporter systems for amino acids were found to be the most abundant peptides from SAR11 [3], suggesting that they were able to take up amino acid. Not surprisingly, based on their known abundance in the wild and the high proportion of ABC transporter in *Roseobacter* and SAR11 bacteria genomic data, the ABC transport proteins were the most frequently detected proteins at such high abundance in marine environments. Hence, the difference in the expressed transporter profiles in marine environments is primarily caused by the geographic distribution of the dominant bacteria population. However, the sampling or analysis method influenced the results of membrane proteomics [5]. The peptide search in the metaproteomic study in the Sargasso Sea revealed only the environmental protein-coding sequences from SAR11 and cyanobacteria [3].

A previous study indicated that relatively abundant bacteria cannot dominate the consumption of all DOMs and that a diverse assemblage of bacteria is essential for the complete degradation of complex DOM in the oceans [50]. The abundant operational taxonomic unit belongs to the members of SAR11, *Roseobacter*, cyanobacteria, CFB group, and *Gammaproteobacteria* (such as OMG and *Alteromonadales*) across the GOS sites [56]. The major types of high-affinity carbohydrate and amino acid transporters known in the *Roseobacter* and SAR11 bacteria include ABC systems [3], [4]. In contrast, CFB group bacteria, such as *Polaribacter* sp. MED152, possess relatively few transporters for free amino acids and lack carbohydrate-specific ABC transporters [57]. The *Shewanella* species (order *Alteromonadales*) carried TBDTs for GlcNA or chito-oligosaccharides transport across the outer membrane and a specific permease for GlcNA transport, but lacked GlcNA-specific PTS or ABC systems in the cytoplasmic membrane [58]. Thus, a broad array of different substrate utilization patterns for *Gammaproteobacteria* and CFB bacteria in the marine environment could be assumed to occur ubiquitously because of the expressions of TBDTs, although these bacteria possibly represented a lower percentage of overall microbial community nutrient acquisition compared with the high abundance of ABC transporters in pelagic microorganisms, such as SAR11, *Roseobacter*, and cyanobacteria.

In conclusion, transporter sequence information, such as diversity and substrate specificity, provide useful and relevant clues for the DOM utilization of a bacterium, bacterial group, or even a microbial community. In this study, TBDTs for DOM transportation were found to be abundant across marine environments. Based on the prevalence of TBDT and ABC transporter sequence data, the major bacterial groups were found to have distinct DOM uptake patterns. Such a metagenomic analysis can potentially contribute to the understanding of the molecular bases of bacterial activities in the ocean, particularly by highlighting the contributions of *Gammaproteobacteria* and CFB group bacteria with TBDTs to the overall ecosystem function.

## Materials and Methods

### Data Preparation

The NR database (3.8 GB, 12,061,831 sequences; 4,118,133,053 total letters) of protein sequences was downloaded from the National Center for Biotechnology Information (NCBI), and the predicted proteomes of *Citromicrobium bathyomarinum* JL354 and *Citromicrobium* sp. JLT1363 from our laboratory were compared with them. The amino-acid sequences and the site metadata (sample location and environment conditions were listed in Table S3) derived from 57 samples in the GOS [30] expedition were retrieved from MG-RAST (version 2) [59]. The GSa in the GOS data sets was excluded from analysis because it was a suspected contaminant in samples from that site [35]. Comparison of the functional annotation of the metagenomics from the GOS was performed using the tools provided in MG-RAST with a maximal e-value of 1e-5 and a minimal alignment length >100. DNA sequences matching “Ton and Tol transport systems” and “ABC transporter”were exported. The ‘Ton and Tol transport systems’ sequences were translated to create a ‘GOS Ton and Tol protein’, using BLASTX [36] against the GOS AA sequence database (*E*-value threshold 1e-10). Sequence searches for transporters in the bacterial genome were carried out using BLASTP [36] (*E*-value threshold 1e-3) and a custom-made database comprising the members of various transporter families based on the Transporter Classification system [60]–[62]. NCBI annotations of the resulting proteins were scanned manually to remove non-transporter proteins.

### Identification of TBDTs

NCBI-NR protein sequence searches for TBDTs were carried out using HMMER hmmsearch [63] with Pfam hidden Markov models (PF00715 for the N-terminal plug domain and PF00593 for the C-terminal membrane-spanning B-barrel domain) and the NR database. Both matched HMM profile sequences were additionally verified by manual inspection and kept for further analysis. Furthermore, NCBI annotations of the resulting proteins were scanned manually to remove sequences that were labeled as spurious. A BLASTP search of the “GOS Ton and Tol protein database” homolog was conducted against a database containing all TBDT proteins at NCBI with a conservative E-value cutoff of 1e-5. All recruited sequences were subjected to verification using reciprocal BLASTP. The 15,905 and 44,073 homologs of TBDT were identified separately in NCBI-NR protein and GOS metagenomics. The genomic organization of the bacterial TBDT genes was visualized on a linear genome map using the Genome2D program [64].

### TBDT Clustering

TBDTs with known substrates were combined with the predicted TBDT homologs. All vs all BLASTP searches were performed for the TBDTs and a Lek clustering algorithm [37], [38] was applied to cluster proteins. An E-value cutoff of 1e-40 for the BLASTP results and a Lek similarity cutoff of 0.6 were used to build gene family clusters. The retrieved predicted TBDT sequences from GOS and from NR were clustered into 783 clusters containing more than two members.

### Hit Normalization

To reconcile the effects of gene size on hit retrieval, the number of hits (Ng) at all GOS sampling sites were size-normalized to the relative average length of the gene (Lx) compared to the length of *recA* from *E*. *coli* K12 (L*recA*: 1,062 bp) using the equation Nn  =  L*recA*/Lx × Ng, where Nn represents normalized hits and Lx is the average length of TBDT genes each cluster or the average length of all genes in a ABC transporter system (data is available at http://www.tcdb.org/ [62]). To remove the bias of average genome size on the sampling of gene from a given metagenomic community, the number of *recA*-normalized hits for six single-copy genes (*recA*, *atpD*, *gyrB*, *dnaK*, *rpoB* and *tufA*) was averaged per site as described previously [65]. The frequency of TBDT andABC transporter genes relative to the number of single-copy gene hits for each site was then calculated as: number of size-normalized gene hits/average number of size-normalized six single-copy gene hits.

### Bacterial Culture

*Citromicrobium bathyomarinum* JL354 and *Citromicrobium* sp. JLT1363 were maintained and precultured aerobically at 28°C with RO medium as previously [66], and stirred at a rate of 160 rpm in the dark. To establish the starvation-induced stationary phase, the feed of medium to the chemostat was switched off at the end of half-maxima optical density (1/2 ODmax) and subsequently changed (inoculum size: 2% v/v) to a nutrient mix with trace elements (1 mL per L medium) and glucose (final concentration 0.5%). The trace element stock solution contained per L: 3.15 g FeCl_3_.6H_2_O, 4.36 g Na_2_-EDTA.2H_2_O, 0.02 mg vitamin B12, 0.08 mg biotin, 0.4 mg vitamin B1, 0.2 mg vitamin B5, 10.0 mg CoCl_2_.6H_2_O, 9.8 mg CuSO_4_.5H_2_O, 22.0 mg ZnSO_4_.7H_2_O, 180.0 mg MnCL_2_.4H_2_O and 6.3 mg Na_2_MoO_4_.2H_2_O. The stationary phase samples were taken after the cell density had reached a constant value. Cells were collected and centrifuged at 10, 000× g for 20 min at 4°C. The samples for further proteomic analysis were frozen in liquid nitrogen and stored at −80°C.

### Protein Extraction and OFFGEL Digestion

Whole cell lysates were prepared as described previously [67]. Cells were washed with 10 mM Tris-HCl, pH 8.0 and lysed in SDT-lysis buffer using a 1∶10 sample to buffer ratio for 5 min at 95°C. Brief sonication was performed to reduce the viscosity of the lysate, which was centrifuged for 5 min at 16,000×g to remove debris. OFFGEL digest was processed with Endoproteinase Lys-C (Roche, Indianapolis, IN, USA) and Trypsin (Promega, Madson, USA). Filter Aided Sample Preparation was used when in-solution digest was carried out as described previously [67] to desalt large amounts of peptide mixtures for OFFGEL separation.

### Protein Characterized by LC-MS/MS

A Finnigan™ LTQ™ linear ion trap MS (Thermo Electron) equipped with an electrospray interface was connected to the LC setup for eluted peptide detection. Data-dependent MS/MS spectra were obtained simultaneously. Each scan cycle consisted of one full MS scan in profile mode followed by five MS/MS scans in centroid mode with the following Dynamic Exclusion™ settings: repeat count 2, repeat duration 30 s, exclusion duration 90 s. Each sample was analyzed in triplicate.

MS/MS spectra were automatically searched against the NR protein database using the BioworksBrowser rev. 3.1(Thermo Electron, San Jose, CA). Protein identification results were extracted from SEQUEST out files with BuildSummary [68]. The peptides were constrained to be tryptic and up to two missed cleavages were allowed.

Carbamidomethylation of cysteines was treated as a fixed modification, whereas oxidation of methionine residues was considered as a variable modification. The mass tolerance allowed for the precursor ions was 2.0 Da and for fragment ions was 0.8 Da. The protein identification criteria were based on Delta CN (≥0.1) and cross-correlation scores (Xcorr, one charge ≥1.9, two charges ≥2.2, three charges ≥3.75. Gene ontology information was assigned for the identified proteins using InterProScan searches implemented in Blast2GO [69].

## Supporting Information

Figure S1 In *Citromicrobium bathyomarinum* JL354 all operons contain the TBDT gene (marked in red).
(TIF)

Figure S2 In *Citromicrobium* sp. JLT1363 all operons contain the TBDT gene (marked in red).
(TIF)

Figure S3 Functional category distribution for the identified proteins in *Citromicrobium bathyomarinum* JL354 based on their annotations in the Gene Ontology (GO) cell component (A), molecular function (B) and biological processes (C) vocabularies.
(TIF)

Figure S4 Functional category distribution for the identified proteins in *Citromicrobium* sp. JLT1363 based on their annotations in the Gene Ontology (GO) cell component (A), molecular function (B) and biological processes (C) vocabularies.
(TIF)

Table S1 Functional classification of TBDTs among sequenced genomes.
(XLS)

Table S2 Functional classification of TBDTs among marine bacterial genomes.
(XLS)

Table S3 The number and frequency of TBDT and ABC transporter sequences each GOS site.
(XLS)

Table S4 A list of expressed proteins identified in proteomic analysis of *Citromicrobium bathyomarinum* JL354.
(XLS)

Table S5 A list of expressed proteins identified in proteomic analysis of *Citromicrobium* sp. JLT1363.
(XLS)
