# Supplementary figures and images for: Distribution and Functions of TonB-Dependent Transporters in Marine Bacteria and Environments: Implications for Dissolved Organic Matter Utilization
Source: PLoS One. 2012 Jul 19;7(7):e41204. doi: 10.1371/journal.pone.0041204 (PMC3400609; doi:10.1371/journal.pone.0041204)

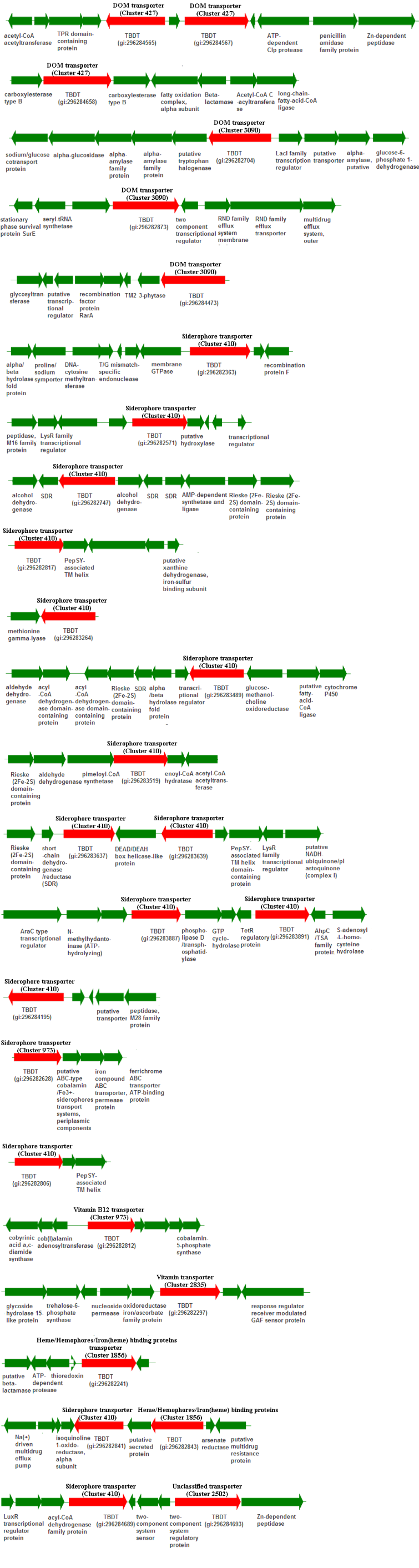

Supplement: Figure S1 — In Citromicrobium bathyomarinum JL354 all operons contain the TBDT gene (marked in red). (TIF) [file pone.0041204.s001.tif]

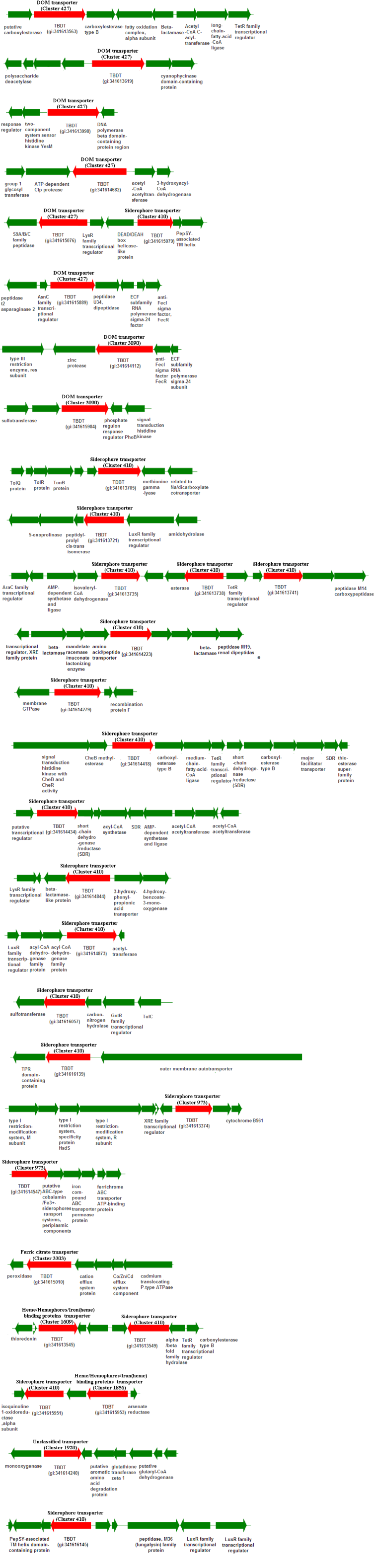

Supplement: Figure S2 — In Citromicrobium sp. JLT1363 all operons contain the TBDT gene (marked in red). (TIF) [file pone.0041204.s002.tif]

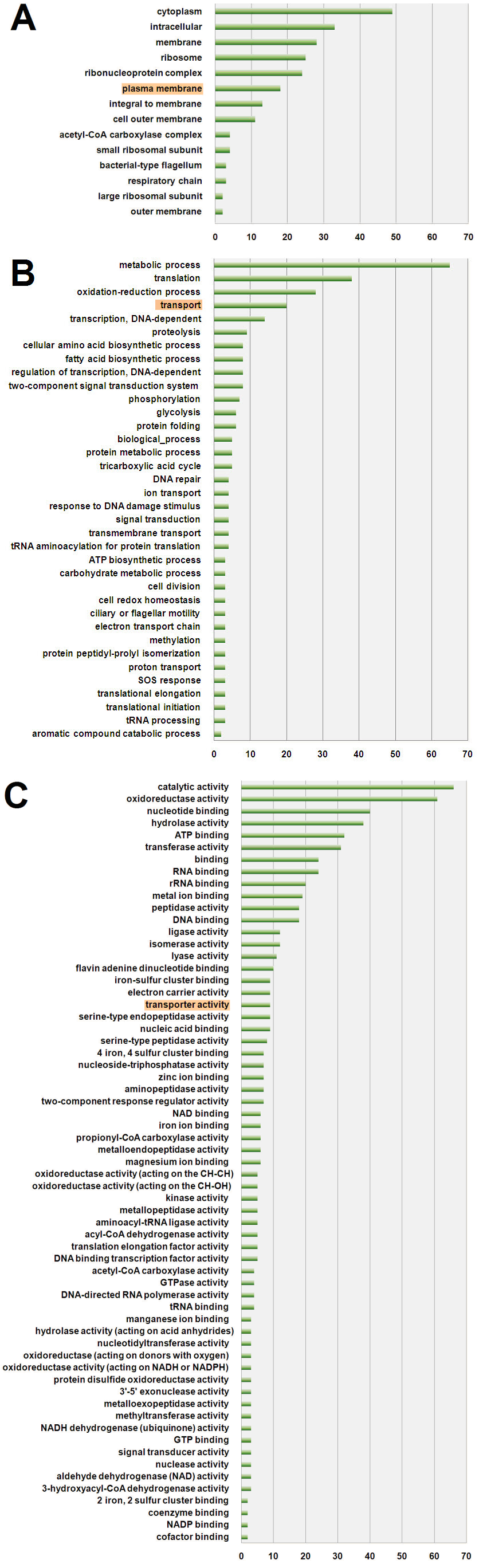

Supplement: Figure S3 — Functional category distribution for the identified proteins in Citromicrobium bathyomarinum JL354 based on their annotations in the Gene Ontology (GO) cell component (A), molecular function (B) and biological processes (C) vocabularies. (TIF) [file pone.0041204.s003.tif]

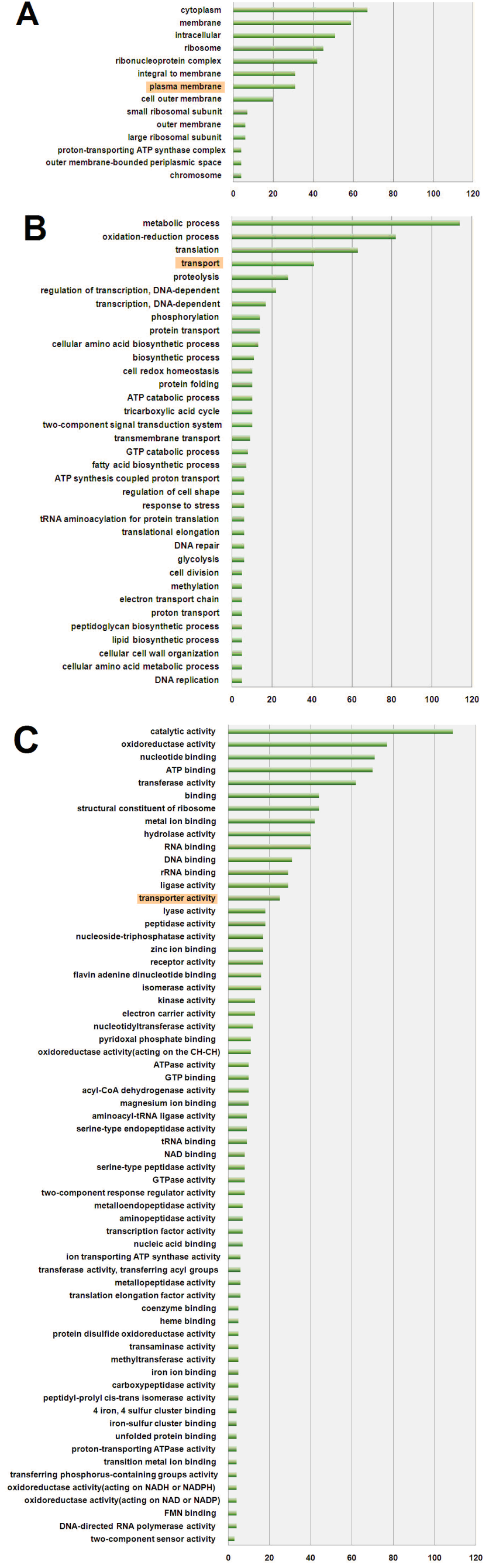

Supplement: Figure S4 — Functional category distribution for the identified proteins in Citromicrobium sp. JLT1363 based on their annotations in the Gene Ontology (GO) cell component (A), molecular function (B) and biological processes (C) vocabularies. (TIF) [file pone.0041204.s004.tif]
